# Under-five anemia and its associated factors with dietary diversity, food security, stunted, and deworming in Ethiopia: systematic review and meta-analysis

**DOI:** 10.1186/s13643-020-01289-7

**Published:** 2020-02-12

**Authors:** Amare Belachew, Tilahun Tewabe

**Affiliations:** College of Medicine and Health Sciences, Bahir Dar University, Bahir Dar, Ethiopia

**Keywords:** Anemia, Prevalence, Determinant, Under-five children, Ethiopia

## Abstract

**Background:**

Anemia is the most common hematologic disorder of children in the globe. There are fragmented and inconclusive study findings on under-five anemia in Ethiopia. Understanding the distribution of anemia is an important step for program planners and policymakers. Therefore, this systematic review was aimed to assess the pooled prevalence of anemia and associated factors with dietary diversity, food security, stunted, and deworming in Ethiopia.

**Methods:**

We searched through African journals of online, Google Scholar, CINHAL, PubMed, Web of Science, Cochrane library, and Scopus. Reviewers used standardized format to extract the data. The data was exported to Stata version 11 software for analysis after extracted by Microsoft excel. The DerSimonian-Laird random-effect model was used to assess the pooled prevalence of under-five anemia. Variation between studies (heterogeneity) was assessed by *I*^2^ statistic test. Publication bias was assessed by the Egger test.

**Result:**

From 561 studies, 16 articles were included in this review. The pooled prevalence of under-five anemia in Ethiopia was 44.83%. In subgroup analysis, the higher pooled prevalence of anemia was observed from children’s age less than 2 years old (50.36%) (95% CI 39.53, 61.18). Poor dietary diversity OR = 1.71 (1.10, 2.68), stunting OR = 2.59 (2.04, 3.28), food insecurity OR = 2.87 (1.25, 6.61), and not dewormed OR = 2.34 (1.77, 3.09) were predictors of under-five anemia.

**Conclusion:**

The magnitude of under-five anemia in this study was extremely high. Therefore, increased coverage of supplementation and fortification programs, periodic deworming, feeding diversified food, supplement food for those who are stunted, and securing food in the households may all alleviate under-five anemia.

## Background

Anemia is the major public health problem and diagnosed as below the reference interval value of hemoglobin or hematocrit concentration for healthy individuals of similar age, sex, and race with similar circumstances [1]. The threshold hemoglobin (Hb) level of under-five children for being anemic is less than 11.0 g/dl [1]. Low consumption and malabsorption of iron-rich foods are the most common causes of under-five anemia [2, 3]. It is prevalent in developing countries and results in poor in cognitive and motor development, low school performances, and exposes comorbid diseases [4].

Under-five anemia is the global health problem both in its severity and prevalence. Globally, 1.6 million people were affected by anemia and 47.4% of them were preschool children [5]. The prevalence of under-five anemia in Ethiopia was 56% [6].

Based on study findings in Ethiopia [7–22], the risk factors of anemia were stunting [7–10], poor dietary diversity [10–13], food insecurity [8, 10–12], timely initiation of complementary feeding [8, 11, 12], deworming [13, 14], wasting [7, 10], educational status [7, 10], maternal weight [7, 9], and antenatal care visits [7]. From these factors, four factors (food diversity, household food security, deworming, and stunting) were sorted for designing adaptable intervention and control strategies to the local context. Additionally, these factors are more prevalent in Ethiopia and given more emphasis regarding the prevention aspects of anemia.

The report of the magnitude of under-five anemia in Ethiopia was not conclusive and consistent. The prevalence of under-five anemia was high in the Somali region (72%) [13], whereas it was low in the Amhara region (13.06%) [21]. There is a high discrepancy among studies and no comprehensive systemic review done on under-five anemia in Ethiopia. This review was conducted to explain and understand the differences in various studies with sex, age, and severity of anemia. Therefore, the purpose of this study was aimed to review sherd evidences regarding the magnitude of under-five anemia in Ethiopia. This study generates epidemiological data in each region of a country and it is important for program planners and policymakers.

## Methods

### Search strategy

The Preferred Reporting Items for Systematic Reviews and Meta-Analyses (PRISMA) guideline was used for this study [23]. Eligible research reports that addressed under-five anemia in Ethiopia were included in this study. Studies published were extensively searched through Google Scholar, CINAHL, PubMed, EMBASE, and Cochrane library. Searching was carried out using the following search terms such as “Anemia”, “Hemoglobin”, “Nutritional deficiency anemia”, Iron deficiency anemia “Under-five children”, “Prevalence”, Magnitude” “Risk factors”, “Predictors”, “Determinants”, “Children”, “Infants”, and “Ethiopia”. Searching words were used in combination and separately by using Boolean operators “OR”, “AND”, “Not”, or combined with this terms.

### Study inclusion

#### Inclusion

Studies done in study designs of cross-sectional, case-control, and cohort reporting prevalence of under-five anemia in Ethiopia were incorporated in this study.

#### Exclusion criteria

Single case study design, qualitative study, research published in books, and research reports not accessed and not written in English were not included.

### Measuring outcome variables

The outcome of this review is under-five anemia and it is diagnosed as hemoglobin (Hb) level is below 11.0 grams per deciliter and it is classified as mild (Hb = 10–10.9 g/dl), moderate (Hb = 7–9.9 g/dl), and severe anemia (Hb less than 7 g/dl) [13]. Secondly, the predictors of under-five anemia were carried out. The determinant factors included in this review were food diversity (poor versus good (poor diet diversity score is defined as children take less than four food groups per day)), deworming (yes versus no), food security (yes versus no), and stunting (yes versus no).

### Quality assessment

The quality of included cross-sectional studies was evaluated with the Newcastle-Ottawa Scale [24]. Two independent reviewers extracted the data. Primary author, publication year, study design, study area, sample size, region of the study, age of participants, level of anemia, sex, and odd’s ratio of determinant factors were included in data extraction formats. The two authors (AB and TT) verified it. Methodological quality, tools deals with the comparability of the study and with the statistical analysis of each original study, and outcome were assessed.

### Statistical analysis

Data were extracted with Microsoft Excel format and exported to the Stata version 11 software for analysis. The random-effect model was used to assess the pooled prevalence of under-five anemia. The variation between studies was quantified by the *I*^2^ statistic test [25]. Age of children was checked with the subgroup analysis. Furthermore, univariate meta-regression analysis was conducted with publication year, severity, sex, study design, region, sample size, and age of children. Fill trill analysis followed by Egger’s tests was used for assessing publication bias [26]. Log odds ratio was used to determine predictors of under-five anemia.

## Results

**Fig. 1:**
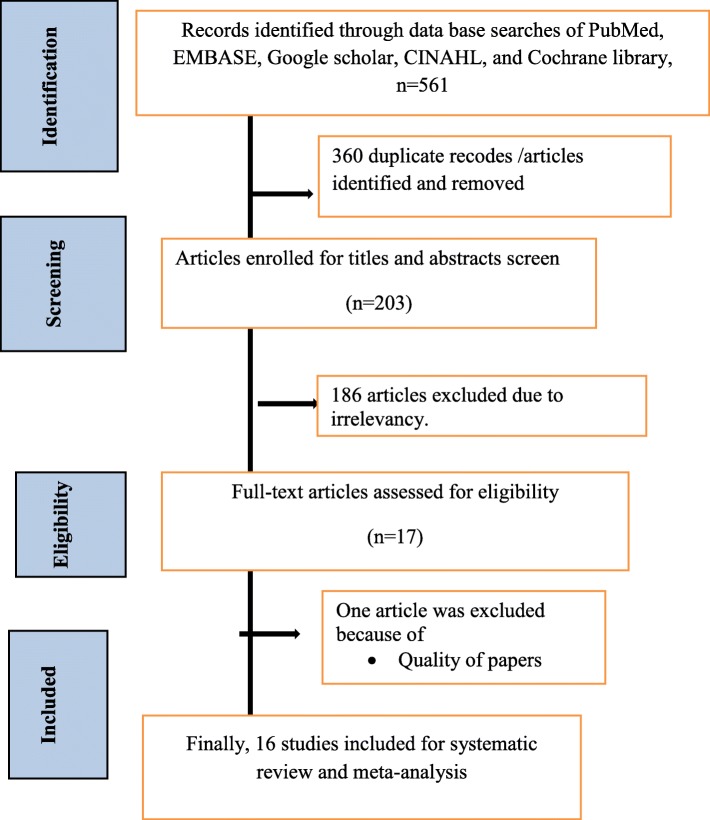
Flowchart diagram of selected studies for systematic review and meta-analysis

A total of 561 research papers that reported the prevalence and associated factors of under-five anemia in Ethiopia were searched by using previous prescribed databases. From the total, 360 research papers were not included due to irrelevancies and duplications. After reviewing of article titles and abstracts, around 184 research papers were excluded. One article was excluded due to quality of the study [27]. Finally, 16 studies were included in this review (Fig. 1).

### Description of included studies

As showed in Table 1, all (16) research included in this systematic review was done by cross-sectional study designs and published from 2013 to 2018. A total of 11,924 under-five children were participated to assess the magnitude of under-five anemia. The included studies showed that the lowest prevalence of under-five anemia was from the Amhara region (13.6%) [21] while the highest prevalence (72%) was from the Somali region [13]. This review includes six studies from Amhara region [8, 14, 16, 19, 21, 22], five from SNNPR [10–13, 20], two from Somali region [9, 17], one from Addis Ababa [15], one from Tigray region [7], and one from Oromia region [18] (Table 1).

**Table 1:** Descriptive summary of 16 studies included in the meta-analysis of the prevalence of anemia among under-five children in Ethiopia, 2018

Authors/year of publication	Region	Study setting	Sample size	Outcome	Prevalence
Gebreziabher and Stoecker, 2017 [15]	Adiss Ababa	Institutional based	150	63	41.7
Alemayehu et al., 2018 [12]	SNNP	Community based	990	650	65.7
Yeshimebet and Selassie, 2016 [20]	SNNP	Institutional based	422	176	41.7
Melako et al., 2018 [11]	SNNP	Community based	485	255	52.6
Tiku et al., 2018 [10]	SNNP	Community based	404	208	
Kawo et al., 2018 [17]	SNNP	Community based	5507	2357	42.8
Abdi Guled et al., 2017 [13]	Somali	Community based	397	286	72
Jemal et al., 2016 [9]	Somali	Community based	399	209	52.4
Gebreegziabiher et al., 2014 [7]	Tigray	Community based	568	212	37.3
Kebede Set al., 2014 [18]	Oromia	Community based	130	43	33.07
Habte et al., 2013 [19]	Amhara	Community based	8260	4155	50.3
Feleke, 2016 [14]	Amhara	Community based	1459	611	41.9
Gashu et al., 2016 [21]	Amhara	Community based	628	82	13.06
Melku et al., 2018 [22]	Amhara	Community based	707	202	28.6
Muchie, 2016 [16]	Amhara	Community based	7636	2184	28.6
Woldie et al., 2016 [8]	Amhara	Community based	347	231	66.6

### Risk of bias, publication bias, and heterogeneity of included studies

**Fig. 2:**
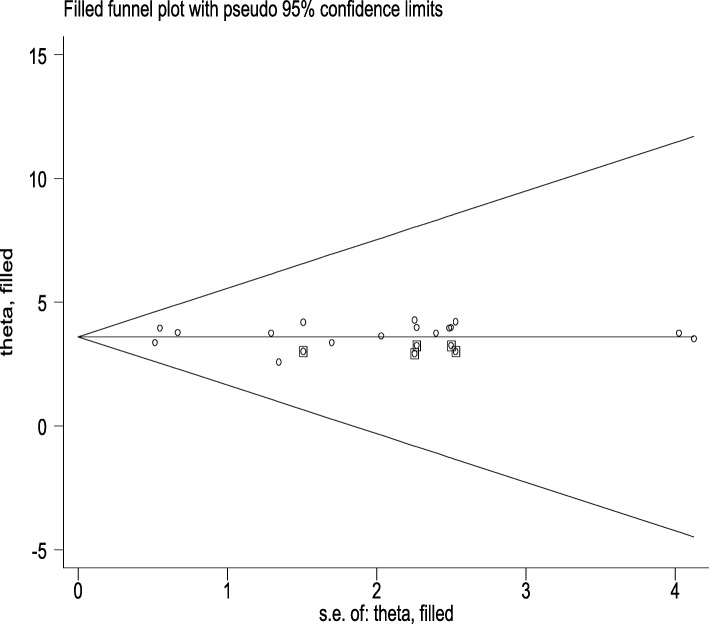
Fill trill analysis of under-five anemia in Ethiopia

The risk of bias assessment tool was conducted to assess risk bias of each study [28]. Finally, summary assessment showed that majority of (75%) included studies had low risk of bias [7–18,], 18.75% had moderate risk of bias [19, 20, 22], and 6.25% had high risk of bias [28]. This review has publication bias as showed by Egger’s test result with a *p* value < 0.001. The Egger BO intercept was 0.075 (− 0.229, 0.378). Fill trill analysis was carried out (Fig. 2). The overall heterogeneity of included studies was *I*^2^ = 95.1% with a *p* value < 0.001, so random-effect model was used to estimate magnitude of under-five anemia.

### Prevalence of under-five anemia in Ethiopia

**Fig. 3:**
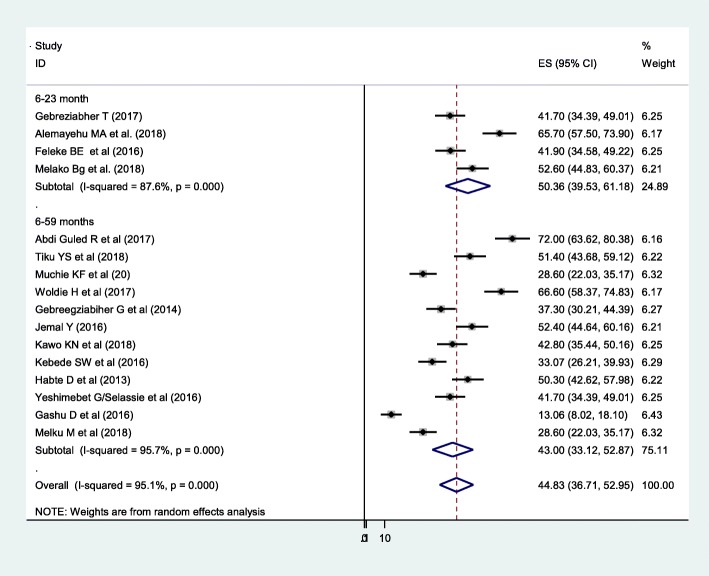
Forest plot of 16 studies on prevalence of anemia among under-five children in Ethiopia

**Fig. 4:**
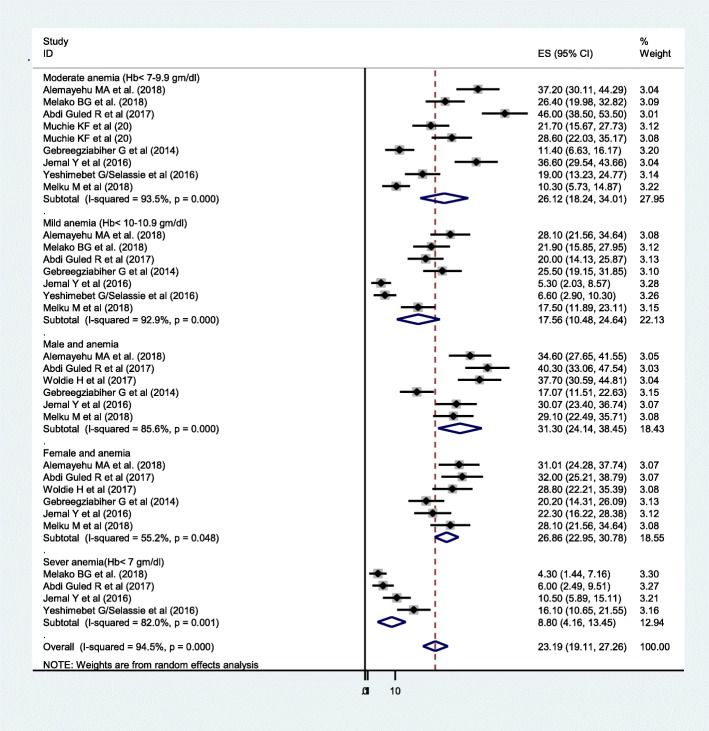
The pooled prevalence of under-five anemia based on severity and sex in Ethiopia

The prevalence of under-five anemia ranges from 13.6% of 628 under-five children in Amhara region [21] to 72% of 397 under-five children in Somali region [13]. The DerSimonian-Laird random-effect model pooled prevalence of under-five anemia in Ethiopia was 44.83% (95% CI 36.71, 52.95). In subgroup analysis, 50.36% of anemia was found in the age range of 6–23 months old and 43% of them were from the age range of 6–59 months old (Fig. 3). The level of severity of anemia in this study was 17.56% (*I*^2^ = 92.9%), 26.12% (*I*^2^ = 93.5%), and 8.8% (*I*^2^ = 82%) had mild, moderate, and severe anemia, respectively. Regarding the sex of the child, anemia was more prevalent in male (31.3%) (*I*^2^ = 85.6%) compared with females (26.86%) (*I*^2^ = 55.2%) (Fig. 4).

#### Meta-regression

Univariate meta-regression was carried out with sample size, publication year, region, and age of children conducted but none of them was significant (Table 2).

**Table 2:** Univariate meta-regression analysis of studies on under-five anemia in Ethiopia, 2018

		Coefficient	*P* value
Year of study		− 0.44	0.988
Sample size		− 0.00066	0.985
Region	Addis Ababa	1	
	Amhara	0.24	0.99
	Oromia	0.33	0.995
	Tigray	− 0.33	0.997
	SNNP	− 0.23	0.996
	Somali	− 0.37	0.991
Age of child	6–23 months old	− 0.376	0.991
	6–59 months old	1	

### Predictors of under-five anemia in Ethiopia

**Fig. 5:**
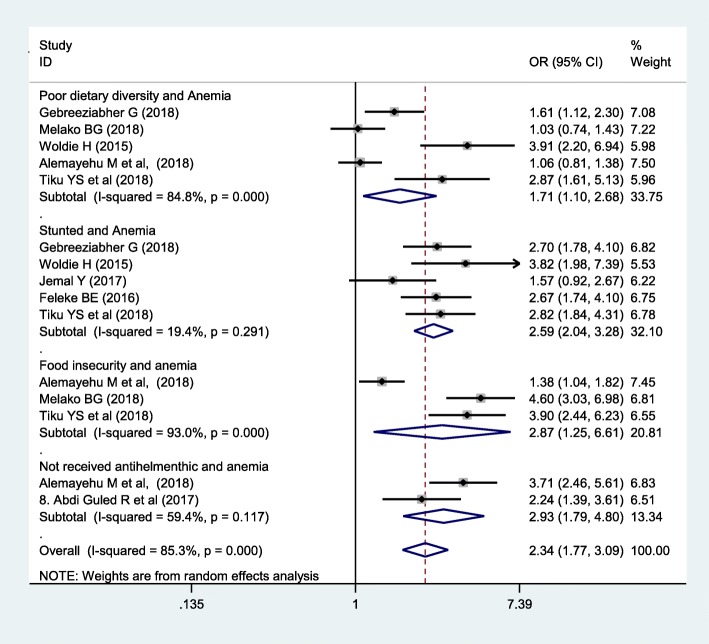
The pooled odds’ ratio of associated factors on under-five anemia in Ethiopia

The association between food security, food diversity, stunting, and deworming with under-five anemia was carried out. In this meta-analysis, to identify the associated factors, five articles were used for poor dietary diversity [7, 8, 10–12], five for stunting [7–10, 14], three for food insecurity [10–12], and two for deworming [12, 13]. Infants who take less than four food groups per day were 1.71 times more likely to have childhood anemia. Infants from food insecure households were 2.87 times more likely to have anemia than their counterparts. The odds ratio of stunted children to develop anemia was 2.54. Infants who were not dewormed were 2.34 times more likely to have anemia than those who received anthelminthic (Fig. 5). Random-effect model was used due to high heterogeneity (*I*^2^ = 85.3% and *p* < 0.1). No publication bias was obtained in Egger’s test (0. 297).

## Discussion

The prevalence of under-five anemia in this review was ranged from 13.06 to 72%. The highest prevalence of under-five anemia was from Somali region [13] while the lowest one was from Amhara region (Gojjam) [21]. The purpose of this review was to assess the pooled prevalence and associated factors of under-five anemia by reviewing the finding of available studies. The pooled prevalence of under-five anemia in Ethiopia was 44.83%. Anemia becomes a public health problem when the magnitude is above 5% of the population [29]. According to World Health Organization (WHO) classifications of anemia, it is severe, moderate, and mild when the prevalence is above 40, 20, 5%, respectively [29]. Thus, the level of under-five anemia in the current study was classified as severe. The findings of the current study is lower than studies done in Cape Verde, West Africa, 51.8% [30] and Tanzania, 84.6% [31]. This could be due to difference in practice of timely initiation of complementary feeding between Ethiopia and Tanzania. In Tanzania, about 84% of children were not exclusively breastfeed [31]. Hence, early introduction of complementary feeding before six-months-old like cow milk should not replace iron-rich foods and which may result in iron deficiency anemia [32].

This result is higher than studies done in China (22.4%) [33], Uganda (37.2%) [34], Colombia (27%) [35], and Denmark (13%) [35]. The possible explanation is in the current study area; there is a high prevalence of hookworm infestation, maternal and childhood malnutrition, malaria infection and a high number of low birth weight babies and stunted children. Additionally, it may be due to variations in socioeconomic status and infant feeding practices (such as exclusive breastfeeding and time of introductions of complementary feeding).

In subgroup analysis, the prevalence of anemia was higher among children under 2 years of age (50.3%) than for children 2–5 years of age (43.3%). This finding is supported by studies done in Ghana [36] and Eastern Cuba [37]. This may be because children born from malnourished mothers have poor stores of iron; infants are more susceptible to infections and diseases that result in poor absorption of iron [38]; the low concentration of iron in breast milk and the introduction of complementary foods often occurs at this age group results high prevalence of anemia compared with children 2–5 years of age.

The finding of this meta-analysis revealed that male under-five children had a higher prevalence of anemia (31.3%) than females (26.8%). This finding is similar to a study done in India [39], but not supported with a systematic analysis of the global anemia burden [40]. This difference could be due to it appears almost entirely driven by the excess prevalence of male anemia resulting from hookworm while excess anemia in females at other ages was related to iron deficiency. Therefore, children should be restricted to barefoot during playing and avoid playing with mud.

In this study, anemia is the major public health problem of the population. The contributing factors for under-five anemia were poor food diversity, food insecurity, stunting, and not dewormed. Children who fed less than four food groups per day were 1.71 times more likely to develop anemia than their counterparts. Similarly, food-insecured children were 2.87 times at high risk to develop anemia than secured ones. This finding is supported in studies done in Italy [41] and middle-income countries [42]. This could be due to children from food insecurity households lack nutritious diets that have high protein quality, adequate micronutrient content and bioavailability, macro-minerals, iron, and essential fatty acids that increase the likelihood of childhood anemia [43].

Children who were stunted were 2.54 times more likely had anemia compared with children who were not stunted. Additionally, children who were not receiving anti-helminthes were 2.34 times more likely to develop anemia than dewormed ones. This finding was consistent with studies done in Tanzania [44], Vietnam [45], Cambodia [46], and Northwest Uganda [47]. This is the general fact that stunting is a consequence of malnutrition and it is a significant risk factor for anemia. Similarly, helminths destroy red blood cells and decrease their lifespan, which is reaching in hemoglobin and finally results in anemia. Therefore, deworming infants every 6 months is the best option of the prevention mechanism of under-five anemia.

## Limitation

Since it is the first systematic review and meta-analysis, it is taken as strength. Predictor variables were estimated by odd’s ratio and it may be affected by other confounding variables. This review has not registered online.

## Conclusion

The pooled prevalence of under-five anemia was classified as severe. Therefore, supplementation and fortification programs of foods, periodic deworming, feeding diversified food, and secured food households are strongly recommended to alleviate under-five anemia.

## Data Availability

Data will be available upon request of the corresponding author.

## Abbreviations

EDHS: Ethiopian Demographic and Health Survey
Hb: Hemoglobin
OR: Odds ratio
WHO: World Health Organization
